# Rewriting the cancer proteome: targeting selective translation as a therapeutic frontier

**DOI:** 10.1172/JCI207335

**Published:** 2026-06-15

**Authors:** Davide Ruggero

**Affiliations:** 1 Helen Diller Family Comprehensive Cancer Center,; 2 School of Medicine and Department of Urology, and; 3 Department of Cellular and Molecular Pharmacology, UCSF, San Francisco, California, USA.

## Abstract

Cancer proteogenomics has revealed that RNA abundance often poorly predicts protein output, highlighting translation as a central determinant of malignant identity. In this issue of JCI, Mishra et al. showed that pharmacologic inhibition of eIF4E cap binding selectively rewired the prostate cancer translatome, suppressing basal keratin translation while promoting luminal features and renewed sensitivity to hormone therapy. More broadly, the study illustrates how tumors exploit selective translation to maintain lineage plasticity, survival, and therapeutic resistance. Targeting translational dependencies may therefore offer a powerful strategy to dismantle cancer-specific proteomic programs and convert resistant cell states into druggable vulnerabilities.

## Translation Defines Malignant Protein Output

One of the most sobering lessons of cancer proteogenomics is that RNA is often a poor proxy for protein. Across tumor studies, mRNA-protein correlations commonly fall in the 0.2–0.5 range, and in a pan-cancer analysis of 1,899 tumors, the median gene-wise correlation was only 0.40, with genes involved in ribosomes and translation among the most weakly coupled (1–7). The mRNA-protein disconnect is not a nuisance variable at the margins of cancer biology, but a central feature of the disease. Malignant identity is not specified by transcript abundance alone; it is enforced by the selective conversion of RNA into protein.

Viruses took advantage of this protein-centered identity principle long before cancer biologists did. They do not need to rewrite the host genome in order to dominate the cell. Instead, viruses hijack the host translational apparatus and privilege a selective RNA pool over the rest (8). Cancer cells appear to exploit a related logic. Under oncogenic transformation, stress, and therapeutic pressure, translation offers a uniquely powerful solution: it can remodel the proteome rapidly, reversibly, and with extraordinary specificity, allowing cancer cells to preserve survival programs and alter lineage state without waiting for a new transcriptional equilibrium (9, 10).

**Figure 1:**
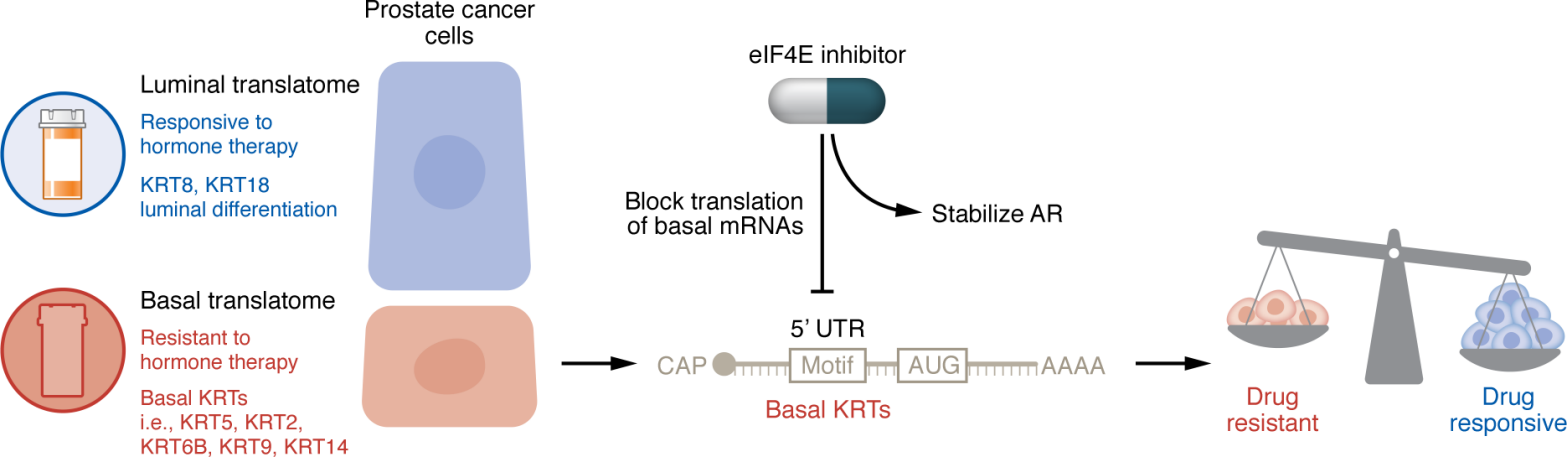
Drugging the cancer translatome. The cancer translatome is a dynamic regulator of malignant cell identity during tumor progression and can profoundly alter therapeutic responses. In prostate cancer, basal subtypes express lower levels of androgen receptor (AR) and exhibit higher resistance to hormone-targeted therapies than luminal subtypes. Recent evidence indicates that pharmacologic inhibition of eIF4E cap binding reshapes the prostate cancer translatome through selective, rather than uniform, suppression of translation. Mishra et al. (17) showed that, in basal-like prostate cancer, eIF4E inhibition preferentially reduced translation of a subset of 5’ UTR–encoded basal keratin mRNAs, including KRT5, KRT2, KRT6B, KRT9, and KRT14. Conversely, inhibition of eIF4E cap-binding activity increased AR protein expression and luminal lineage features, creating a therapeutic vulnerability to anti-AR therapy in resistant tumors. This selective translational vulnerability disrupted basal lineage programs while sparing other mRNA classes, illustrating how transcript-specific control of protein synthesis can be exploited to reprogram tumor cell state and enhance therapeutic sensitivity.

That logic places eIF4E at the center stage of malignant transformation and adaptation. As the cap-binding component of eIF4F, eIF4E controls entry into cap-dependent translation and has long stood at the crossroads of many oncogenic signaling pathways (11, 12). Importantly, genetic work showed that normal mammalian development tolerates reduced eIF4E dosage, whereas cancer cells exploit a reserve of eIF4E activity that exists in excess of what is required for normal tissues (13). Elevated eIF4E does not simply raise translation uniformly; it selectively enhances the production of transcripts encoding for key oncogenes such as Myc as well as growth, survival, angiogenesis, invasion, and stress adaptation, helping explain why eIF4E has repeatedly behaved as a bona fide oncogenic driver (14–16). For this reason, targeting the cancer translatome is emerging as the next frontier in drug discovery (Figure 1). If transcription lays the groundwork for malignant potential, translation may be where malignant fate is finally enforced.

## eIF4E selectivity rewires prostate cancer

In the accompanying manuscript, Mishra and colleagues showed that the eIF4E cap-binding activity transcends its canonical role as a permissive component of translation initiation, acting as a lineage-specifying vulnerability in advanced prostate cancer (17). Using the cap-binding inhibitor PF-07293623 (developed by eFFECTOR Therapeutics and licensed to Pfizer) (18), the authors showed that inhibiting eIF4E suppressed growth across castration-resistant prostate cancer models, with particularly strong effects in androgen receptor–low (AR-low), basal-like disease. Mechanistically, PF-07293623 blocks eIF4E engagement with the m7G cap and lowers overall protein synthesis by roughly 40% (17). Yet, the most important conceptual point is that this global decrease in translation does not affect all transcripts equally.

Indeed, the power of the study lies in PF-07293623’s specificity. Despite a substantial reduction in bulk protein synthesis, the translational output that remains is highly selective. The authors showed that the translation of basal keratin mRNAs was disproportionately repressed by PF-07293623, without concordant decreases in their mRNAs, whereas luminal keratins were comparatively spared (17). Mishra et al. went further by identifying a shared 5′ UTR cis-regulatory element that encodes this selectivity, demonstrating that sensitivity to eIF4E cap-binding inhibition is intrinsically embedded within transcript architecture itself (17). These findings align with prior work in other cancer contexts, showing that eIF4E-dependent translational specificity is dictated by distinct 5′ UTR motifs in mRNAs encoding metabolic and growth-promoting programs, including antioxidative stress pathways (10, 15). In other words, even when overall translation is reduced, the consequence is not a uniform collapse of protein synthesis. Rather, eIF4E-dependent translation reveals a hierarchy of transcript sensitivity that is highly context- and cell-state–specific. This is precisely why translational control is so biologically potent: it is global enough to reallocate proteome output, yet selective enough to privilege particular phenotypes.

The consequence in this setting is a striking rewiring of lineage identity in prostate cancer cells. By repressing a basal keratin program that is not merely diagnostic but functionally required for survival, PF-07293623 began to dismantle the basal state itself (17). At the same time, inhibition of the eIF4E cap-binding activity drove a reciprocal increase in AR protein and luminal features, not through increased AR transcription, but through posttranscriptional stabilization mediated by the deubiquitinases BAP1 and OTUD3 (17). The result was not simply growth inhibition, but cell-state reprogramming: an aggressive, therapy-resistant basal prostate cancer was pushed toward a more luminal and therapeutically vulnerable state. This mechanistic switch becomes clinically meaningful in the in vivo setting, where Mishra et al. showed that PF-07293623 resensitized resistant tumors to enzalutamide (an anti-AR therapy commonly used to treat castration-resistant prostate cancer) and converted lineage plasticity from a driver of resistance into a druggable liability (17).

## Translational Therapies Reveal Tumor Vulnerabilities

Mishra et al.’s findings present a clinical opportunity to control translation more precisely than broadly reducing protein synthesis. PF-07293623’s targeting of eIF4E-dependent translation selectively dismantles the proteomic program on which tumors depend. Another compelling example comes from zotatifin, an eIF4A inhibitor that has shown encouraging early clinical activity in heavily pretreated estrogen receptor–positive (ER-positive) metastatic breast cancer, including durable regressions in some patients and a favorable tolerability profile (19). Notably, zotatifin was recently demonstrated to have a remarkable antitumor activity in preclinical models of prostate cancer through its selective suppression of the translation of oncogenic drivers such as AR and other growth-promoting factors (20). These translation-targeted agents reinforce the concept that translation can be targeted with specificity by disrupting the expression of key oncogenic transcripts. Taken together, these studies suggest that translation-targeted therapies need not function as blunt cytotoxic agents. When matched to the correct translational dependency, they can selectively collapse a malignant translatome while preserving a usable therapeutic window. That idea is clinically important because it reframes translation from a generic housekeeping process into an actionable layer of tumor specificity.

## Mapping future translational dependencies

The next step for the field is to define the rules of translational vulnerability with far greater precision. An important consideration in targeting translation is the potential for toxicity, given its essential role in normal cellular physiology. However, both genetic and pharmacological evidence support the existence of a therapeutic window. Here, partial reduction of eIF4E activity was well tolerated in vivo yet markedly impaired tumorigenesis, indicating that cancer cells are selectively dependent on elevated eIF4E-driven translation (17), as previously shown in ref. 13. A similar principle has been observed with targeting eIF4A, where both preclinical and early clinical studies demonstrate antitumor activity with manageable toxicity (19, 20). This differential sensitivity likely reflects the reliance of tumors on the enhanced translation of specific oncogenic and stress-response mRNAs, whereas normal tissues are less sensitive to modest perturbations in translation.

We need to know which transcript features, i.e., sequence motifs, RNA structures, upstream open reading frames, cap-dependence thresholds, and cooperating RNA-binding proteins, determine whether a given mRNA falls, escapes, or even rises when canonical initiation is constrained. We also need to map these dependencies with translatome-resolved approaches directly in patient tumors, ideally integrating ribosome profiling, proteomics, and spatial analyses so that lineage state, translational state, and therapeutic response can be understood together rather than in isolation. Just as importantly, the most effective translation therapies will probably not be blunt monotherapies. Their greatest value may lie in rational combinations, where they collapse resistant cell states and resensitize tumors to hormone therapy, targeted therapy, or immunotherapy.

An important implication of this work is that eIF4E selectivity is unlikely to be determined by cap binding alone. Increasing evidence suggests that eIF4E functions within a broader regulatory network that includes RNA-binding proteins, cis-acting RNA structural features, cap-proximal RNA modifications, and dynamic interactions with the initiation machinery itself. Prior work has shown that eIF4E-sensitive translatomes are enriched for distinct 5’ UTR signatures, that RNA-binding proteins such as LARP1 can directly compete with eIF4E at the cap to regulate specific transcript classes, that cap-adjacent m6Am can alter cap-dependent translation and eIF4E engagement, and that eIF4F cooperates structurally with helicases and ribosome-associated factors during recruitment and scanning (13, 15, 21–23). Defining the network of eIF4E-interacting partners that shapes this selectivity will therefore be especially important. Identifying these cofactors and understanding how they cooperate with eIF4E to regulate distinct translational programs could uncover new layers of control over oncogenic mRNA translation and may reveal additional therapeutic targets within these regulatory complexes.

Mishra et al.’s study therefore opens a broader agenda for the future. Which tumors are truly addicted to eIF4E-controlled translatomes? What biomarkers — for example, high eIF4E expression, basal lineage features, specific 5’ UTR elements, or distinct proteomic states — will best identify the patients most likely to benefit? How generalizable is this vulnerability across basal-like cancers in different tissues? And what resistance mechanisms will emerge once the cancer cell is forced to route around cap-dependent control? These questions define the path by which the field will move from elegant mechanistic insight to durable therapeutic strategy.

Mishra et al. provide a persuasive answer to a question that has lingered over the translation field for years: can selective control of mRNA translation regulate lineage fate in cancer in a way that is both mechanistically coherent and therapeutically useful? Their data suggest that the answer is yes. More broadly, the work sharpens a larger idea whose time has come. Cancer does not simply hijack transcriptional programs; it hijacks the machinery that decides which RNAs become protein. The cancer translatome is therefore not a passive readout of transformation but an active engine of malignant identity, and, increasingly, a targetable one (17).

## Conflict of interest

DR was a cofounder of eFFECTOR Therapeutics, is an SAB member at SJP Biotec, and MelliCell.

## Funding support

This work is the result of NIH funding, in whole or in part, and is subject to the NIH Public Access Policy. Through acceptance of this federal funding, the NIH has been given a right to make the work publicly available in PubMed Central.

- The National Institutes of Health (grants R35CA242986, R01CA271437).
- American Cancer Society (American Cancer Society Research Professor Award).

## Version 1 06/15/2026

Electronic publication
